# Perceived HIV knowledge in the context of novel psychoactive substance use: evidence from a multi-city survey in Kazakhstan

**DOI:** 10.3389/fpubh.2026.1774432

**Published:** 2026-03-10

**Authors:** Botagoz Turdaliyeva, Gulshara Aimbetova, Venera Baisugurova, Gulzar Shah, Nargiza Yussupova, Manshuk Ramazanova, Anastassiya Minina, Sultan Seidumanov, Indira Karibayeva

**Affiliations:** 1 Kazakh Scientific Center of Dermatology and Infectious Diseases, Department of Nursing, Asfendiyarov Kazakh National Medical University, Almaty, Kazakhstan; 2 Department of Public Health, Asfendiyarov Kazakh National Medical University, Almaty, Kazakhstan; 3 Department of Biostatistics and Foundations of Scientific Research, Asfendiyarov Kazakh National Medical University, Almaty, Kazakhstan; 4 Department of Health Policy and Community Health, Jiann-Ping Hsu College of Public Health, Georgia Southern University, Statesboro, GA, United States; 5 Kazakh Scientific Center of Dermatology and Infectious Diseases, Almaty, Kazakhstan; 6 Department of Public Health and Social Sciences, Kazakhstan Medical University “KSPH”, Asfendiyarov Kazakh National Medical University, Almaty, Kazakhstan; 7 Department of Nursing, Asfendiyarov Kazakh National Medical University, Almaty, Kazakhstan

**Keywords:** Kazakhstan, novel psychoactive substances, NPS use, perceived HIV knowledge, sexual behavior, socio-demographic factors, substance use

## Abstract

**Background and aim:**

This study aims to investigate perceived HIV knowledge among NPS users in Kazakhstan and evaluate patient characteristics associated with higher perceived HIV knowledge.

**Methods:**

We conducted an applied, cross-sectional survey of NPS users in Kazakhstan between March and October 2024. The dependent variable, perceived HIV knowledge, was modeled as an ordinal outcome using proportional-odds ordinal logistic regression. All analyses were conducted in R using RStudio.

**Results:**

Substantial heterogeneity in perceived HIV knowledge was observed across regions, behaviors, and substance-use patterns. Participants from Petropavl (AOR = 3.24) and Shymkent (AOR = 1.61) were significantly more likely to demonstrate higher perceived HIV knowledge compared to those from Astana. Sexual behavior characteristics showed mixed associations: inconsistent condom use during paid sex was linked to lower knowledge (never vs. every time: AOR = 0.49; sometimes vs. every time: AOR = 0.35), whereas reporting loss of control during sex (AOR = 1.07) and engaging in anal sex with non-regular partners in the past 3 months (AOR = 2.12) were positively associated with perceived HIV knowledge. Substance-use patterns also showed significant associations: polydrug injection increased the likelihood of higher perceived HIV knowledge (AOR = 2.27). At the same time, both recent and non-recent syringe sharing were inversely associated (AOR = 0.14 and 0.15, respectively).

**Conclusion:**

Our findings emphasize that perceived HIV knowledge is not evenly distributed and is shaped by complex interactions between geography, sexual behavior, and substance use. Therefore, effective interventions for this population must be comprehensive—combining access to substance use treatment, sexual health education, harm reduction programming, psychological support, and stigma reduction.

## 1 Introduction

Globally, Human Immunodeficiency Virus (HIV) remains a significant public health challenge. According to surveillance data from the World Health Organization (WHO), an estimated 40.8 million people were living with the virus in 2024, with over 1.3 million new infections reported that year (1). Despite significant progress in prevention and treatment, knowledge gaps persist among key populations, particularly those engaged in high-risk behaviors such as substance use. Among these emerging risks is the use of novel psychoactive substances (NPS)—synthetic compounds designed to mimic the effects of traditional illicit drugs such as cannabis, cocaine, ecstasy, or LSD, while circumventing legal restrictions (2). Examples include synthetic cannabinoids (“spice”), synthetic cathinones (“bath salts”), and designer opioids (3–5). In 2021, the European Monitoring Centre for Drugs and Drug Addiction was tracking 880 NPS compounds, compared to 560 in 2015, reflecting the accelerated expansion of this drug category (6). Although NPS use has been linked to increased vulnerability to HIV transmission and worse mental health outcomes (7), it remains underexamined in the context of HIV knowledge and prevention, especially in the local context.

In Kazakhstan, HIV incidence is mainly concentrated among people who inject drugs and other marginalized groups, such as men who have sex with men (8). A recent prediction study estimates that the prevalence of HIV infection in Kazakhstan will rise from 0.29% in 2021 to 0.47% by 2030 (9). The country has also witnessed a rise in NPS use, particularly among younger populations in urban centers. A study shows that the prevalence of NPS use varied across regions, accounting for approximately 10% at the national level in 2018 (10). Another study found that during the COVID-19 pandemic, the odds of polydrug and NPS use were 1.6 times higher among younger individuals, with elevated odds observed across all age groups (11). Additionally, national statistics show that in 2021, 2022, and 2023, 69.4, 74.8, and 73.8% of synthetic drug trafficking offenses, respectively, were committed by youth aged 18 to 34 (12). Notably, parenteral HIV transmission and intravenous drug use remain disproportionately higher among men (13). Despite these trends, empirical data on HIV knowledge among NPS users remain limited, and few studies have examined the socio-demographic, structural, and behavioral correlates of HIV knowledge within this population.

Understanding HIV knowledge requires an understanding of the intersectionality of social and economic conditions with behaviors and beliefs, particularly in contexts shaped by novel psychoactive substance use. Previous studies show that social determinants of health (SDoH), such as education level, employment, homeownership, and income, impact HIV knowledge and behaviors (14, 15). The SDoH, however, have a synergetic effect through intersection with behavioral and psychosocial factors to affect people’s knowledge about HIV infection and how they avoid it. These socioeconomic disadvantages increase the risk of reliance on peer networks where certain beliefs about transmission prevail, thereby making people less likely to perceive the actual benefits of preventive measures (16). On the other hand, having more money can help fill knowledge gaps by making it easier to conduct private testing, implement digital prevention efforts, and engage healthcare experts who help people better understand their risk (17).

Existing literature suggests that HIV knowledge is a critical determinant of preventive behavior, yet its distribution is shaped by intersecting factors such as education, income, housing instability, and access to care (18, 19). Studies in similar settings have shown that individuals with lower socioeconomic status and limited health service utilization are less likely to possess accurate information about HIV transmission and prevention (20, 21). Moreover, sexual risk behaviors—such as inconsistent condom use, chemsex, and non-consensual encounters—have been associated with both low HIV knowledge and elevated transmission risk (22, 23). While these associations have been documented in broader substance-using populations, the specific dynamics among NPS users in Kazakhstan remain poorly documented and understood. No prior studies have specifically assessed perceived HIV knowledge among NPS users in Kazakhstan, nor more broadly in the Eastern Europe and Central Asia (EECA) region. Addressing this gap is particularly urgent in this context, where both HIV incidence and synthetic drug use are on the rise.

This study is grounded in a conceptual framework informed by the Social Determinants of Health (SDoH) model and the Health Belief Model (HBM). According to these frameworks, perceived HIV knowledge is shaped by multiple, intersecting predictors. The HBM posits that health behaviors are influenced by perceived susceptibility, severity, benefits, and barriers, all of which are mediated by an individual’s perceived knowledge and self-efficacy (24, 25). In turn, the SDoH model emphasizes that structural and contextual inequalities shape varying access to accurate information and services (26). In this study, we conceptualize perceived HIV knowledge not only as an outcome influenced by these domains but also as a potential mediator of future HIV-preventive behavior.

This study aims to investigate perceived HIV knowledge among NPS users in Kazakhstan and evaluate socio-demographic, structural, sexual-behavior, and substance-use characteristics associated with higher perceived HIV knowledge.

## 2 Materials and methods

### 2.1 Study design and study population

We conducted an applied, cross-sectional survey of NPS users in Kazakhstan between March and August 2025. The study was conducted in three urban centers representing distinct geographic regions: Shymkent (South Kazakhstan), Petropavl (North Kazakhstan), and Astana (capital). Participants were recruited from clients engaged in HIV prevention programs and undergoing testing at regional Centers for HIV prevention and non-governmental organizations (NGOs). NPS users were defined as individuals who answered “yes” to a screening question regarding recent NPS use at the time of recruitment and were actively receiving HIV prevention services. NPS use was self-reported and referred to the consumption of synthetic cannabinoids, cathinones, or other designer substances intended to mimic the effects of controlled drugs. Recruitment followed a non-probability snowball sampling strategy, starting with individuals already accessing HIV prevention services. These initial participants were invited to refer peers within their networks who also used NPS. No financial or material incentives were provided for participation.

Data were collected exclusively using paper-based questionnaires, with anonymity and voluntary informed consent. To ensure ethical rigor and effective engagement with key populations, trained outreach workers were mobilized in each region. These individuals had established access to and trust within target communities. Outreach workers responsible for recruitment were trained by a team of addiction specialists and public health researchers to ensure standardized procedures across sites. The training covered ethical considerations, inclusion criteria, confidentiality protocols, and appropriate engagement strategies for hard-to-reach populations. Outreach workers provided detailed explanations of the study’s purpose and procedures, obtained verbal and written consent, and were present during questionnaire completion to offer clarification and support.

The target sample size was 750 participants, with 250 recruited from each city. This sample size was calculated based on a multivariable logistic regression framework, using the rule-of-thumb of at least 10 outcome events per predictor variable. The study included 24 groups of independent variables spanning socio-demographic, structural, sexual-behavioral, and substance-use domains. Assuming that approximately 33% of participants would report high perceived HIV knowledge—based on the distribution across three ordinal categories (“not informed,” “somewhat informed,” and “well informed”)—the minimum required sample size was estimated as:


$$n=\frac{10\times 24}{0.33}\approx 727$$

To account for potential missing data, non-response, and the need for subgroup analyses across geographic regions and behavioral risk profiles, the sample size was rounded up to 750. This approach ensured adequate statistical power for both descriptive and multivariable analyses, while maintaining feasibility across the three study sites.

Eligibility criteria (inclusion): (1) current NPS user; (2) age 18–44 years; (3) any sex; (4) fluency in Russian or Kazakh; and (5) written informed consent. Exclusion criteria: minors (<18 years), pregnant women, those aged>45 years, and those who declined consent.

The study protocol adhered to ethical principles for research with human participants; all respondents provided informed consent prior to enrollment. The local ethics committee of Kazakhstan’s Medical University “KSPH” approved this study under IRB-134-2024, dated 19.12.2024.

### 2.2 Instrument development

The questionnaire used in this study was adapted from the validated Questionnaire for Assessing Behavioral Health Risks among People Who Inject Drugs and Members of Their Social Networks, originally developed for HIV behavioral surveillance in key populations (27). The instrument was selected due to its relevance to substance use, sexual risk behaviors, and structural vulnerabilities commonly observed among NPS users.

To ensure contextual appropriateness, the original tool was reviewed by a multidisciplinary panel of experts in HIV epidemiology, behavioral science, and harm reduction in Kazakhstan. Sections pertaining to tuberculosis co-infection and incarceration history were excluded from the final version, as they were not directly aligned with the study’s objectives. Additional items were incorporated to capture NPS-specific patterns, chemsex behaviors, and perceived HIV transmission risk, based on formative interviews with outreach workers and program staff from regional AIDS Centers and NGOs. The finalized questionnaire was translated from Russian to Kazakh using a forward-backward translation protocol, with discrepancies resolved by consensus among bilingual public health professionals. Importantly, the adapted questionnaire was formally registered as a copyrighted literary work under the title “Questionnaire for Studying the Risks of HIV Transmission among Users of Novel Psychoactive Substances (NPS)” on March 3, 2025 (Certificate no. 55574) (28).

The questionnaire primarily comprised single-item measures of self-reported behaviors, many of which are standard indicators used in national surveillance and outreach programs. Unlike latent psychological constructs (e.g., depression, stigma), these items do not require formal psychometric validation. They are designed to be easily understood by participants without specialized knowledge, capturing observable behaviors such as condom use, drug administration methods, and frequency of testing.

The questionnaire underwent cognitive testing with a subsample of 30 NPS users in Astana to assess clarity, cultural relevance, and comprehension. Minor revisions were made to improve item phrasing and response options. The questionnaire was administered in paper format under conditions of anonymity and informed consent. The instrument was designed to be completed within 20–25 min and was piloted prior to full deployment to ensure feasibility in field settings.

### 2.3 Dependent and independent variables

The primary outcome was perceived HIV knowledge, assessed using a self-reported measure based on the question: “How well-informed do you feel about the risk of HIV infection?” Responses were categorized into three levels: “not informed,” “somewhat informed,” and “well informed.” For analysis, perceived HIV knowledge was modelled as an ordered outcome (lowest to highest knowledge: not informed < somewhat informed < well informed). In descriptive analyses, we summarize group distributions; in regression models, we interpret effects as odds of being in a higher perceived HIV-knowledge category.

Independent variables were measured using a structured, predominantly closed-ended questionnaire developed for this study. The instrument included an introductory script (purpose, confidentiality, contact information), a brief socio-demographic section, and modules aligned with the survey objectives: structural factors, sexual behavior characteristics, and substance use domains. Socio-demographic factors included sex (female, male), age in years (continuous), marital status (divorced/widowed, not married, married), education (bachelor’s degree or higher, some college, high school, did not complete high school), residency (rural, urban), and region (Astana, Petropavl, Shymkent). Structural characteristics comprised living conditions (cohabitation with non-relatives/homeless/other, renting, owning, cohabiting with relatives), primary income source (begging/other, wages, social payments/support, illegal income, family/friends/partner), employment status (full-time, part-time, unemployed), monthly income level [<50,000 KZT (equivalent of 100 USD); 50,000–100,000 KZT; 100,000–200,000 KZT, ≥200,000 KZT], detention history (no, yes), and health-service utilization in the past year (public outpatient, hospital, private outpatient, HIV-prevention centers, and NGO services; each coded yes/no). Sexual-behavior measures included number of sexual partners (count; treated as non-normally distributed), paid sex in the past 3 months (for money/drugs or for food/stay; each coded yes/no), condom use during paid sex (every time, sometimes, never), anal sex with a non-regular partner in the past 3 months (yes/no), sex with (rather than without consent) in the past 3 months (yes/no), perceived partner HIV risk in the past 3 months (yes/no), chemsex use (yes/no), perceived drug use–sexual risk association (yes/no), and reported barriers to consistent condom use, including loss of control, no thoughts on HIV risk, impossibility to buy condoms nearby, lack of money, unwillingness to show fear, and partner unwillingness (each coded yes/no). Substance-use variables included injection drug use (yes/no) and specific drug patterns relative to no use—marijuana, heroin injection, heroin inhalation/peroral, methadone injection, methadone inhalation/peroral, psychostimulant injection, psychostimulant inhalation/peroral, polydrug injection, and other—coded as binary indicators.

### 2.4 Statistical analysis plan

All analyses were prespecified and conducted using R, version 4.3.2 (2023-10-31) (29). We first performed data quality checks and verified coding of ordered categories. Because the survey used non-probability (snowball) recruitment, estimates are interpreted as associations within the study sample rather than population prevalence, and no sampling weights were applied.

Continuous variables were summarized as mean ± standard deviation (SD) when approximately normally distributed and as median (IQR; range) otherwise; categorical variables were summarized as n (%) by HIV-knowledge category. Group differences were assessed using Pearson’s *χ*^2^ tests for categorical variables (reporting *χ*^2^ and *p*). For continuous variables, we evaluated normality and homoscedasticity; if the assumptions were met, we used a one-way ANOVA; otherwise, we used the Kruskal–Wallis test.

The dependent variable, perceived HIV knowledge, was modeled as an ordinal outcome (three ordered levels) using proportional-odds (cumulative logit) ordinal logistic regression. All assumptions of the proportional-odds model (POM) were checked and met, supporting the appropriateness of the proposed analysis. We adopted a two-stage strategy within conceptual domains: (1) univariable screening of each candidate predictor within its domain (socio-demographic, structural, sexual-behavior, and substance-use), followed by (2) domain-specific multivariable models including screened predictors and a parsimonious set of *a priori* covariates. In univariable screening, we controlled the family-wise error rate using a Bonferroni correction across the number of tests in the domain; variables with *p <* 0.0017 advanced to multivariable modeling. Final models report adjusted odds ratios (AORs) with 95% confidence intervals and two-sided *p*-values (*α* = 0.05). Significant adjusted associations from domain models were visualized as forest plots with AORs on a log scale and 95% CIs, color-coding factors associated with higher odds (AOR > 1, green) versus lower odds (AOR < 1, red).

## 3 Results

### 3.1 Description of the study participants

Table 1 presents the distribution of socio-demographic, structural, sexual-behavior, and substance-use characteristics stratified by perceived HIV knowledge categories among the overall analytic sample (*N* = 750). Several contrasts were statistically significant and clinically meaningful. Among the socio-demographic characteristics, gender, age, education, and residency (urban/rural) distributions were similar across groups. Marital status differed (*χ*^2^ = 11.5, *p* = 0.021), with smaller proportions married among the well-informed (8.5%) than among the not-informed (17.2%). Knowledge differed sharply by region (*χ*^2^ = 79.1, *p <* 0.001). Participants classified as not informed were disproportionately from Astana (63.3%) and much less often from Petropavl (8.6%), whereas those well informed were most commonly from Petropavl (42.1%) and least from Astana (25.3%). Additional details about the study participants are provided in Supplementary Table S1.

**Table 1:** Patient characteristics by perceived HIV knowledge.

Characteristic		Not informed, *n* (%)	Somewhat informed, *n* (%)	Well informed, *n* (%)	*χ* ^2^	*p*
Socio-demographic characteristics						
Gender	Male	102 (79.7)	158 (79.4)	347 (82)	0.8	0.682
	Female	26 (20.3)	41 (20.6)	76 (18)		
Age (Mean ± SD)		37.0 ± 10.0	33.9 ± 8.2	36.7 ± 9.2	6.3 (*F*-value)	0.307
Marital status	Not married	68 (53.1)	108 (54.3)	242 (57.2)	11.5	0.021
	Married	22 (17.2)	32 (16.1)	36 (8.5)		
	Divorced/widowed	38 (29.7)	59 (29.6)	145 (34.3)		
Education	Did not complete HS	11 (8.6)	32 (16.1)	67 (15.8)	7.9	0.247
	HS	43 (33.6)	67 (33.7)	156 (36.9)		
	Some college	58 (45.3)	83 (41.7)	155 (36.6)		
	Bachelor’s and higher	16 (12.5)	17 (8.5)	45 (10.6)		
Residency	Urban	121 (94.5)	185 (93)	392 (92.7)	0.5	0.767
	Rural	7 (5.5)	14 (7)	31 (7.3)		
Region	Astana	81 (63.3)	62 (31.2)	107 (25.3)	79.1	<0.001
	Petropavl	11 (8.6)	61 (30.7)	178 (42.1)		
	Shymkent	36 (28.1)	76 (38.2)	138 (32.6)		

HS, High School; HIV, human immunodeficiency virus; NGO, non-governmental organizations; SD, standard deviation.

### 3.2 Ordinal logistic regression results

In univariable proportional-odds models including the socio-demographic covariates, region was the strongest correlate of higher perceived HIV knowledge: compared with Astana (reference), respondents from Petropavl had over fourfold higher odds (OR = 4.13, 95% CI 2.88–5.94; *p <* 0.001) of being in a higher knowledge category and those from Shymkent had approximately twofold higher odds (OR = 2.01, 95% CI 1.43–2.81; *p <* 0.001). No univariable associations were observed for gender, age, marital status, education, or residency at *α* = 0.0017. In the multivariable model, region remained independently associated with perceived HIV knowledge: Petropavl (AOR = 3.24, 95% CI 2.16–4.87; *p <* 0.001) and Shymkent (AOR = 1.61, 95% CI 1.12–2.31; *p* = 0.011) both had higher adjusted odds of being in a higher knowledge category relative to Astana.

Supplementary Table S2 presents structural characteristics associated with the perceived HIV knowledge. No univariable associations were observed for living conditions, income source, employment, income level, detention history, or use of health services (Table 2).

**Table 2:** Factors associated with perceived HIV knowledge: univariable and multivariable ordinal regression results of socio-demographic characteristics.

Characteristic		OR (95% CI)	*p*-value	AOR (95 CI)	*p*-value
Gender (Ref. Female)	Male	1.16 (0.82–1.64)	0.413		
Age		1.02 (1.00–1.03)	0.049		
Marital status (Ref. Divorced/widowed)	Not married	0.93 (0.68–1.27)	0.637		
	Married	0.49 (0.31–0.77)	0.002		
Education (Ref. Bachelor’s and higher)	Did not complete HS	1.31 (0.74–2.33)	0.352		
	HS	1.11 (0.67–1.82)	0.685		
	Some college	0.86 (0.53–1.41)	0.554		
Residency (Ref. Rural)	Urban	0.84 (0.48–1.46)	0.542		
Region (Ref. Astana)	Petropavl	4.13 (2.88–5.94)	<0.001	3.24 (2.16–4.87)	<0.001
	Shymkent	2.01 (1.43–2.81)	<0.001	1.61 (1.12–2.31)	0.011

AOR, adjusted odds ratio; HIV, Human Immunodeficiency Virus; OR, odds ratio; Ref, reference.

Table 3 presents sexual behavior characteristics associated with the perceived HIV knowledge. In univariable proportional-odds models, several sexual-behavior indicators were related to perceived HIV knowledge. A higher number of sexual partners was associated with greater odds of being in a higher knowledge category (OR per partner = 1.12, 95% CI 1.07–1.18; *p <* 0.001). Compared with always using condoms during paid sex, reporting sometimes (OR = 0.60, 95% CI 0.42–0.88; *p* = 0.001) or never (OR = 0.42, 95% CI 0.29–0.61; *p <* 0.001) was associated with lower odds of higher knowledge. Anal sex with a non-regular partner (last 3 months) was associated with higher knowledge (OR = 3.31, 95% CI 2.09–5.23; *p <* 0.001). Additional univariable associations were observed for perceived partner HIV risk (OR = 2.70, 95% CI 1.71–4.25; *p <* 0.001), chemsex use (OR = 1.95, 95% CI 1.47–2.58; *p <* 0.001), and perceived drug use–sexual risk association (OR = 2.11, 95% CI 1.53–2.92; *p <* 0.001) versus no. Among reported barriers to consistent condom use, loss of control showed higher odds (OR = 3.14, 95% CI 2.32–4.25; *p <* 0.001); other barriers were not statistically significant. In the multivariable model adjusting for the sexual-behavior covariates, four variables retained independent associations with perceived HIV knowledge. Each additional sexual partner remained positively associated (AOR = 1.07, 95% CI 1.01–1.13; *p* = 0.028). Relative to always using condoms during paid sex, reporting sometimes (AOR = 0.35, 95% CI 0.22–0.55; *p <* 0.001) or never (AOR = 0.49, 95% CI 0.32–0.74; *p <* 0.001) was associated with lower odds of higher knowledge. Anal sex with a non-regular partner remained positively associated (AOR = 2.38, 95% CI 1.41–4.01; *p* = 0.001). Reporting loss of control as a barrier to condom use also remained associated with higher knowledge (AOR = 2.12, 95% CI 1.41–3.20; *p <* 0.001). Associations observed in univariable analyses for perceived partner HIV risk, chemsex use, and perceived drug use–sexual risk association did not persist after adjustment.

**Table 3:** Factors associated with perceived HIV knowledge: univariable and multivariable ordinal regression results of sexual behavior characteristics.

Characteristic		OR (95% CI)	*p*-value	AOR (95 CI)	*p*-value
Number of sexual partners		1.12 (1.07–1.18)	<0.001	1.07 (1.01–1.13)	0.028
Paid sex (last 3 months) (Ref. No)	For money and drugs	1.43 (0.95–2.16)	0.083		
	For food and stay	3.21 (1.46–7.03)	0.004		
Condom use behavior for paid sex (Ref. Every time)	Sometimes	0.60 (0.42–0.88)	0.001	0.35 (0.22–0.55)	<0.001
	Never	0.42 (0.29–0.61)	<0.001	0.49 (0.32–0.74)	<0.001
Anal sex with not a regular partner (last 3 months) (Ref. No)	Yes	3.31 (2.09–5.23)	<0.001	2.38 (1.41–4.01)	0.001
Sex with consent (last 3 months) (Ref. No)	Yes	1.76 (0.93–3.33)	0.084		
Perceived partner HIV risk (last 3 months) (Ref. No)	Yes	2.70 (1.71–4.25)	<0.001	1.31 (0.77–2.71)	0.322
Chemsex use (Ref. No)	Yes	1.95 (1.47–2.58)	<0.001	0.99 (0.67–1.48)	0.972
Perceived drug use-sexual risk association (ref. No)	Yes	2.11 (1.53–2.92)	<0.001	1.21 (0.80–1.83)	0.356
Reported barrier to consistent condom use (Ref. No)	Loss of control	3.14 (2.32–4.25)	<0.001	2.12 (1.41–3.20)	<0.001
	No thoughts on HIV risk	1.55 (1.10–2.16)	0.011		
	Impossible to buy condoms nearby	0.53 (0.28–0.98)	0.044		
	Lack of money	0.67 (0.27–1.66)	0.393		
	Unwillingness to show fear	1.66 (0.83–3.31)	0.150		
	Partner unwillingness	1.60 (0.93–2.77)	0.090		

AOR, adjusted odds ratio; HIV, Human Immunodeficiency Virus; OR, odds ratio; Ref, reference.

Table 4 presents substance use behavior characteristics associated with the perceived HIV knowledge. In univariable proportional-odds models, several substance use behavior indicators were related to perceived HIV knowledge. Injection drug use (yes vs. no) was not associated. Across the drug-use pattern indicators (reference: no use), no associations were observed for marijuana, heroin injection, heroin inhalation/peroral use, methadone injection, methadone inhalation/peroral, and psychostimulant injection. Two univariable signals were observed: psychostimulant inhalation/peroral was associated with lower odds of higher knowledge (OR = 0.56, 95% CI 0.39–0.80; *p* = 0.001) compared to no psychostimulant inhalation or peroral use; and polydrug injection was associated with higher odds of higher perceived HIV knowledge (OR = 2.82, 95% CI 1.53–5.19; *p <* 0.001) compared to no polydrug use. By recency of shared syringe use (reference: >1 month ago), both recent sharing (OR = 0.15, 95% CI 0.07–0.34; *p <* 0.001) and never sharing (OR = 0.15, 95% CI 0.09–0.27; *p <* 0.001) showed associations compared to more than a month ago. The history of sharing a syringe with a known HIV-positive person was not associated in univariable analysis. In the multivariable model of substance-use characteristics, recency of shared syringe use remained independently associated with perceived HIV knowledge: recent sharing (AOR = 0.14, 95% CI 0.06–0.34; *p <* 0.001) and never sharing (AOR = 0.15, 95% CI 0.08–0.28; *p <* 0.001), each relative to more than a month ago. Polydrug injection was also associated with two-fold odds of higher perceived HIV knowledge (OR = 2.27, 95% CI 1.18–3.45; *p <* 0.001) compared to no polydrug use. No other substance-use indicators retained statistically significant adjusted associations. This multivariable model explains about 27.9% of the variance in the ordinal outcome.

**Table 4:** Factors associated with perceived HIV knowledge: univariable and multivariable ordinal regression results of substance use behavior characteristics.

Characteristic		OR (95 CI)	*p*-value	AOR (95 CI)	*p*-value
Injection drug use (Ref. No)	Yes	1.22 (0.92–1.63)	0.164		
Drug use pattern (Ref. No)	Marijuana use	0.83 (0.62–1.10)	0.190		
	Heroin injection	0.80 (0.58–1.10)	0.164		
	Heroin inhalation / peroral	1.40 (0.56–3.48)	0.476		
	Metadon injection	1.11 (0.66–1.88)	0.699		
	Metadon inhalation/peroral	0.98 (0.40–2.41)	0.965		
	Psychostimulators injection	0.97 (0.59–1.58)	0.899		
	Psychostimulators inhalation/peroral	0.56 (0.39–0.80)	0.001	1.10 (0.69–1.45)	0.681
	Polydrug injection	2.82 (1.53–5.19)	<0.001	2.27 (1.18–3.45)	<0.001
	Other				
Recency of shared syringe use (unsterilized) (Ref. More than a month ago)	Recently	0.15 (0.07–0.34)	<0.001	0.14 (0.06–0.34)	<0.001
	Never	0.15 (0.09–0.27)	<0.001	0.15 (0.08–0.28)	<0.001
History of shared syringe use with a known HIV-positive person (Ref. No)	Yes	1.89 (1.06–3.37)	0.031		
Nagelkerke R^2^ = 0.279					

AOR, adjusted odds ratio; HIV, Human Immunodeficiency Virus; OR, odds ratio; Ref, reference.

**Figure 1:**
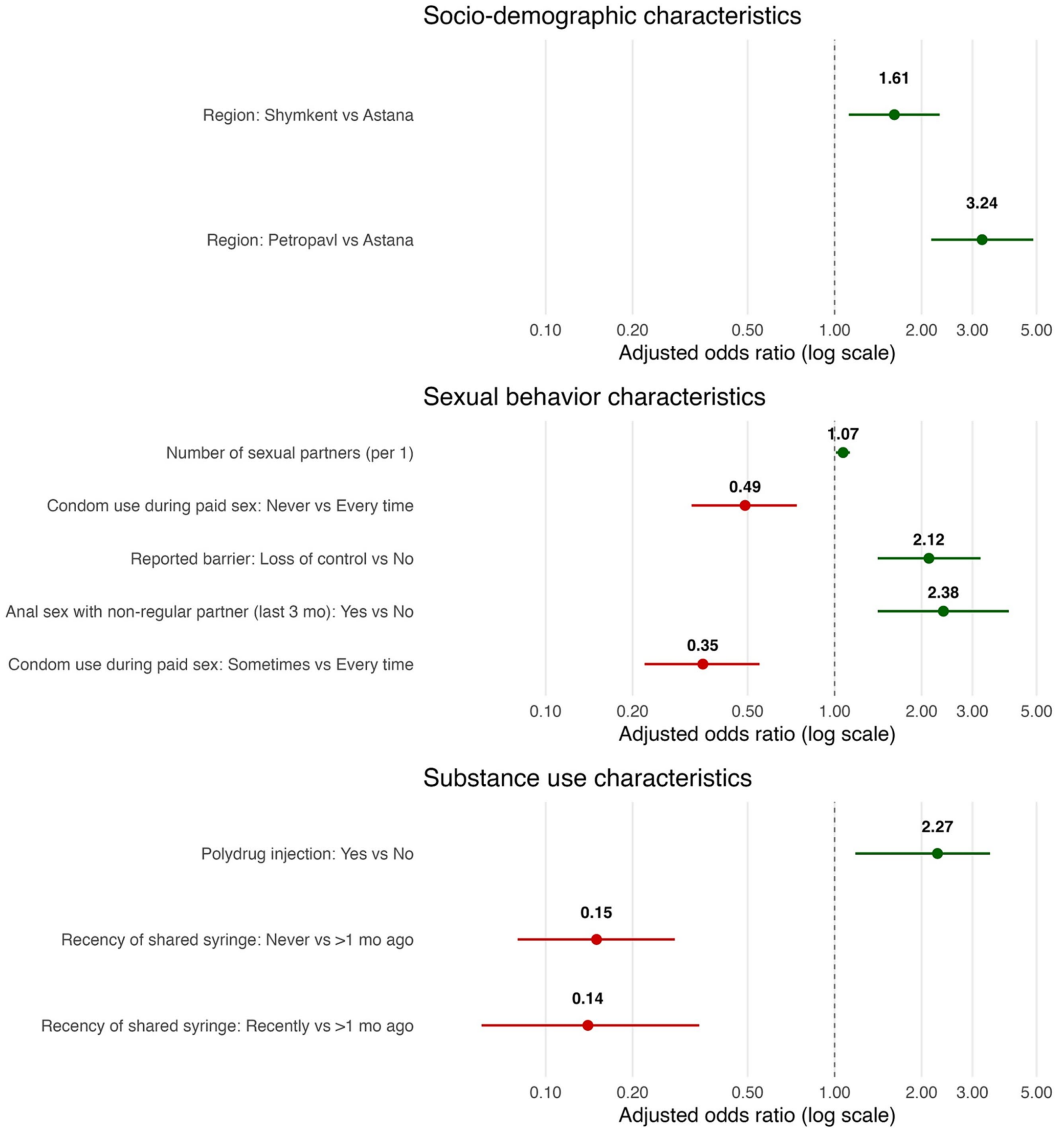
Forest plot of adjusted associations with perceived HIV knowledge.

Figure 1 presents the significant factors associated with higher levels of perceived HIV knowledge across all domains examined. Variables linked to increased odds of greater perceived HIV knowledge are highlighted in green, while those associated with decreased odds are shown in red.

## 4 Discussion

This study sought to investigate perceived HIV knowledge among NPS users in Kazakhstan and to identify socio-demographic, structural, sexual-behavioral, and substance-use characteristics associated with higher levels of perceived HIV knowledge. Our findings reveal substantial heterogeneity in perceived HIV knowledge across geographic regions, behavioral profiles, and substance-use patterns. Notably, participants from Petropavl and Shymkent were significantly more likely to be well-informed compared to those from Astana. Sexual risk behaviors—including inconsistent condom use, anal sex with non-regular partners, and chemsex—were strongly associated with perceived HIV knowledge levels, as were substance-use patterns such as polydrug injection and recency of syringe sharing. These results affirm the study’s central hypothesis: perceived HIV knowledge among NPS users is shaped by intersecting behavioral and structural factors, and not merely by individual-level knowledge or education.

Perceived knowledge remains meaningful even when it does not perfectly align with objective knowledge. Individuals who believe they are well-informed often show greater confidence in engaging with prevention services. They also demonstrate stronger risk perception and higher motivation to adopt protective behaviors (30, 31). In contrast, low perceived knowledge can reduce engagement, testing uptake, and self-efficacy in negotiating safer practices (32, 33). In contexts of substance use, where impulsivity and stigma undermine prevention, perceived knowledge functions as a psychological resource shaping willingness to act on available information (32, 33). These dynamics align with the HBM, which emphasizes that health behaviors are driven by perceived susceptibility, severity, benefits, barriers, and self-efficacy. Perceived knowledge directly informs these constructs by influencing how individuals interpret risk, confidence, and the value of prevention. As a result, it becomes a critical determinant of HIV-related behavior beyond factual knowledge alone (25, 33).

To the best of the authors’ knowledge, this is the first study to assess perceived HIV knowledge among NPS users in Kazakhstan. Our findings align with a growing body of literature indicating that perceived HIV knowledge is a critical determinant of preventive behavior. Yet, its distribution is uneven across populations with overlapping vulnerabilities. Prior studies in Central Asia and in Kazakhstan have documented similar disparities in HIV knwodege among people who inject drugs (PWID), particularly in contexts marked by criminalization, stigma, and limited access to care (34–36). The 2024 national HIV report by the Kazakh Scientific Center of Dermatology and Infectious Diseases highlights a rise in NPS use, which is associated with an increase in chemsex practices and a rapid clustering of sexually transmitted HIV infections (37). Engaging NPS users poses significant challenges for outreach workers in harm reduction programs. At the same time, effective prevention and treatment strategies for synthetic drug dependence remain limited (37). Interestingly, the paradoxical finding that individuals reporting non-consensual sex and higher partner counts were more likely to be well informed suggests that knowledge alone may not be sufficient to mitigate risk in contexts of coercion or structural vulnerability. This paradox may reflect a process of reactive learning, where individuals with greater risk exposure acquire more knowledge through repeated contact with outreach or clinical services. However, this increased knowledge does not always translate into safer behavior due to entrenched behavioral patterns, emotional trauma, or limited self-efficacy in high-risk contexts (38, 39).

Safe behavior among people who use psychoactive substances is a multifaceted challenge involving psychological, social, and structural dimensions. Beyond disparities in perceived HIV knowledge, stigma remains a pervasive barrier—particularly within healthcare settings. A recent survey found that 87% of healthcare providers in Kazakhstan agreed with at least one statement indicative of stigmatizing attitudes (40). Surveys conducted in 2015 and 2020 using the HIV Stigma Index among people living with HIV (PLHIV) in Kazakhstan consistently revealed that healthcare facilities were the most frequently cited environments for experiencing stigma and discrimination. These facilities surpassed other social or institutional settings in reported stigma (41). This persistent stigma has profound implications for HIV knowledge, access to care, and testing uptake (20, 42). When individuals anticipate judgment, discrimination, or breaches of confidentiality, they are less likely to seek HIV-related services, even when they possess adequate knowledge (43). Stigma not only erodes trust in providers but also reinforces internalized shame. This further distances key populations from prevention and treatment pathways (44, 45). In our study, the paradoxical finding that those reporting higher-risk sexual behaviors were also more informed may reflect a survival-driven engagement with information. However, knowledge alone does not guarantee linkage to care or sustained health-seeking behavior.

Moreover, emerging evidence on best practices in NPS harm reduction suggests that NPS use is not solely a public health or legislative issue. It is also deeply embedded in cultural and economic contexts (46). Building trust with NPS users and promoting informed, safer use subcultures are essential components of effective harm-reduction strategies (46). Behavioral interventions must go beyond information dissemination to address the psychological and social drivers of risk. These include impulsivity, loss of control, and diminished decision-making capacity under the influence of stimulants. Even individuals who are aware of HIV transmission risks may disregard protective measures during intoxication or withdrawal. These dynamics underscore the need for integrated, trauma-informed approaches that combine substance use treatment, sexual health education, and stigma reduction.

Although gender, employment status, and income level were not independently associated with perceived HIV knowledge in the multivariable analysis, their descriptive differences warrant reflection. Male participants were slightly more represented in the well-informed group. This may reflect greater engagement in risk behaviors that prompt exposure to HIV education through harm reduction outreach. Similarly, observed differences in employment and income may indirectly reflect disparities in service access or exposure to educational interventions. Those with more economic stability may be more connected to formal services. From the perspective of the Social Determinants of Health framework, these variables—such as gender, employment status, and income—are structural conditions that influence access to health information, services, and risk environments (47). Their influence may be mediated through behavioral pathways, shaping opportunities for engagement with HIV prevention efforts. However, these relationships were not statistically significant in adjusted models. This suggests that more proximal behavioral and contextual exposures, rather than baseline socio-economic status, are more critical determinants of perceived HIV knowledge in this population.

The marked differences in perceived HIV knowledge across cities—particularly the lower levels observed in Astana—warrant further exploration. One contributing factor may be the more substantial presence of community-based harm reduction programs in Petropavl and Shymkent. In these cities, local NGOs offer more consistent access to education, prevention tools, and peer support. In contrast, Astana may rely more heavily on institutional services that are less embedded within high-risk networks. Regional differences in stigma, service accessibility, and engagement with marginalized groups may also shape exposure to HIV-related messaging. Additionally, national data from 2024 indicate that HIV prevalence among adults aged 15–49 is 0.34% in Astana, 0.59% in Petropavl, and 0.25% in Shymkent. This suggests that higher levels of perceived knowledge in areas like Petropavl may reflect a more visible or pressing local epidemic. Such visibility may prompt greater investment in prevention and education efforts (48). Future research should explore these city-level differences through qualitative or implementation-focused studies to better understand how local service environments influence perceived HIV knowledge outcomes.

These geographical disparities may also reflect differences in how local health systems engage with key populations and deliver outreach-based education. Global Fund and UNAIDS reports note that community-based harm reduction and peer-led education initiatives are more consistently implemented in northern and southern Kazakhstan, whereas Astana relies more heavily on institutional services with limited penetration into high-risk networks (49, 50). These regional disparities in service coverage may contribute to the spatial differences in perceived HIV knowledge observed in our study.

These findings carry important implications for HIV prevention programming in Kazakhstan. First, they underscore the need for regionally tailored interventions that account for local epidemiological and behavioral profiles. The elevated odds of perceived HIV knowledge among participants from Petropavl and Shymkent suggest that community-based outreach and harm reduction efforts in Kazakhstan regions may offer scalable models for replication. Second, the strong associations between sexual risk behaviors and perceived HIV knowledge highlight the importance of integrating sexual health education into substance-use treatment and outreach services. Programs should prioritize not only information dissemination but also empowerment strategies that address barriers such as loss of control and access to condoms. Third, the data suggest that individuals engaged in polydrug use and recent syringe sharing may represent critical touchpoints for intervention. This is particularly relevant given their elevated risk and variable knowledge levels. Future studies should explore the causal pathways linking substance-use patterns to HIV knowledge and prevention behaviors, ideally through longitudinal designs or implementation trials. Importantly, stigma must be addressed as a central barrier to the success of any intervention. To achieve the UNAIDS 95–95-95 targets (49) and advance toward the global goal of HIV elimination, Kazakhstan must invest in stigma-reduction strategies at every level of the health system. This includes training healthcare providers in nonjudgmental, trauma-informed care, expanding peer-led outreach models, and embedding anti-stigma messaging into public health campaigns.

This study has several limitations. The use of a non-probability snowball sampling design, initiated within HIV prevention services, likely led to an overrepresentation of individuals already engaged in care, who may be more informed about HIV risks compared to the broader population of NPS users. Furthermore, recruiting participants through existing HIV prevention services may also influence the accuracy of self-reported behaviors because participants connected to services may be more aware of socially desirable responses, potentially underreporting high-risk behaviors due to social desirability bias. This introduces selection bias and limits the external validity of the findings. All estimates should be interpreted as associations within the sampled group, not as prevalence estimates. Self-reported data may be subject to recall bias or social desirability bias, particularly regarding sensitive behaviors such as paid sex and syringe sharing. The cross-sectional design precludes causal inference, and the reliance on a single global item to assess perceived HIV knowledge may not capture the full spectrum of understanding. The use of a single-item measure to assess perceived HIV knowledge limits evaluation of internal consistency and comparability with studies using validated multi-item indices. This choice, however, was deliberate given the challenges of engaging hard-to-reach populations, where lengthy instruments could reduce response quality or participation. A concise format ensured feasibility, comprehension, and higher response rates in anonymous outreach settings. Future research should incorporate validated scales to strengthen psychometric analysis and enable international comparison. Finally, the recall periods varied slightly across items, which may introduce measurement inconsistencies across participants and cities. Nonetheless, the study benefits from methodological strengths, including a robust sample size, regionally diverse recruitment, and domain-specific multivariable modeling. The use of trained outreach workers and anonymous data collection likely enhanced data quality and participant trust.

## 5 Conclusion

This study provides novel insights into the behavioral and structural correlates of perceived HIV knowledge among NPS users in Kazakhstan. Our findings emphasize that HIV knowledge is not evenly distributed and is shaped by complex interactions between geography, sexual behavior, and substance use. Even individuals who are well-informed about HIV transmission may disregard protective measures under the influence of substances. This behavioral volatility illustrates that knowledge alone is a necessary but insufficient condition for risk reduction. A complex interplay of biological vulnerability, mental health, stigma, and access to harm reduction services drives risk practices. Therefore, effective interventions for this population must be comprehensive—combining access to substance use treatment, sexual health education, harm reduction programming, psychological support, and stigma reduction. These results matter because they identify actionable gaps in prevention programming and suggest that targeted, regionally responsive interventions may be more effective than one-size-fits-all approaches. We recommend that future research prioritize longitudinal designs to assess changes in perceived HIV knowledge over time and evaluate the impact of integrated harm reduction and sexual health interventions. Policymakers and practitioners should consider these findings when designing outreach strategies, allocating resources, and developing culturally competent messaging for key populations.

## Data Availability

The datasets presented in this article are not readily available because the HIV-related data used in this study contain sensitive information and were collected exclusively for the purposes of this research under institutional ethics approval. In accordance with IRB requirements, the data will be securely retained and destroyed 3 years after publication of the authorized research articles. Access to the data is restricted; however, reasonable and well-justified requests may be considered by the corresponding author, subject to approval by the local ethics committee of Kazakhstan’s Medical University. Requests to access the datasets should be directed to BT, turdaliyeva@kncdiz.kz.

## References

[1] World Health Organization (WHO). HIV data and statistics. Fact Sheets. (2025):1–8.

[2] Ritchie H Arriagada P Roser M. Opioids, cocaine, Cannabis, and other illicit drugs. Our World in Data. (2022)

[3] Tracy DK Wood DM Baumeister D. Novel psychoactive substances: types, mechanisms of action, and effects. BMJ. (2017) 356. doi: 10.1136/BMJ.I6848 28122697

[4] Hohmann N Mikus G Czock D. Effects and risks associated with novel psychoactive substances: Mislabeling and Sale as Bath salts, spice, and research chemicals. Dtsch Arztebl Int. (2014) 111:139. doi: 10.3238/ARZTEBL.2014.0139 24661585 PMC3965957

[5] Ventura L Carvalho F Dinis-Oliveira RJ. Opioids in the frame of new psychoactive substances network: a complex pharmacological and toxicological issue. Curr Mol Pharmacol. (2017) 11:97–108. doi: 10.2174/1874467210666170704110146/CITE/REFWORKS 28676005

[6] Galvin B. European Drug Report, 2022. Drugnet Ireland. (2022):1–36. https://www.drugsandalcohol.ie/37104/1/Drugnet_Ireland_Issue_82.pdf

[7] Dal Farra D Valdesalici A Zecchinato G De Sandre A Saccon D Simonato P . Knowledge and use of novel psychoactive substances in an Italian sample with substance use disorders. Int J Environ Res Public Health. (2022) 19:915. doi: 10.3390/IJERPH19020915/S1 35055743 PMC8776073

[8] Davlidova S Haley-Johnson Z Nyhan K Farooq A Vermund SH Ali S. Prevalence of HIV, HCV and HBV in Central Asia and the Caucasus: a systematic review. Int J Infect Dis. (2021) 104:510–25. doi: 10.1016/j.ijid.2020.12.068 33385583 PMC11094609

[9] Mussina K Kadyrov S Kashkynbayev A Yerdessov S Zhakhina G Sakko Y . Prevalence of HIV in Kazakhstan 2010-2020 and its forecasting for the next 10 years. HIV/AIDS - Research and Palliative Care. (2023) 15:387–97. doi: 10.2147/HIV.S413876 37426767 PMC10329475

[10] Prilutskaya M Yussopov O Negay N Altynbekov K Tokayeva M. Prevalence of new psychoactive substances addiction: a hospital-based cross-sectional study. Journal of Clinical Medicine of Kazakhstan. (2020) 1:11–6. doi: 10.23950/1812-2892-JCMK-00730

[11] Akkuzinova K Inoue K Toleuov E Moldagaliyev T Seksenbayev N Jamedinova U . Differences in the rates of diagnoses of mental and Behavioral disorders due to psychoactive substance use by sex and age during pre-pandemic and COVID-19 pandemic periods in Kazakhstan. Healthcare. (2024) 12:2012. doi: 10.3390/HEALTHCARE12202012 39451427 PMC11506856

[12] Prilutskaya M Mankieva V. “Synthetic drug issues in Kazakhstan: emphasising youth and women’s involvement “In: Stöver H Michels II, editors. New psychoactive substances. Baden-Baden, Germany: Nomos Verlagsgesellschaft mbH & Co. KG (2025). 147–62. doi: 10.5771/9783748943204-147

[13] Bilibayeva G Ospanova D Nurkerimova A Kussainova F Tukeev M Shokybaeva M . Epidemiological analysis of HIV/AIDS in Kazakhstan during 2018-2020. J Res Health Sci. (2023) 23:e580–11. doi: 10.34172/jrhs.2023.115 PMC10422132 37571951

[14] Waterfield KC Shah GH Etheredge GD Ikhile O. Consequences of COVID-19 crisis for persons with HIV: the impact of social determinants of health. BMC Public Health. (2021) 21:299. doi: 10.1186/S12889-021-10296-9 33546659 PMC7863613

[15] Jennings Mayo-Wilson L Peterson SK Kiyingi J Nabunya P Sensoy Bahar O Yang LS . Examining cash expenditures and associated HIV-related Behaviors using financial diaries in women employed by sex work in rural Uganda: findings from the Kyaterekera study. Int J Environ Res Public Health. (2023) 20:5612. doi: 10.3390/IJERPH20095612 37174132 PMC10178413

[16] Richner DC Lynch SM. Sexual health knowledge and sexual self-efficacy as predictors of sexual risk Behaviors in women. Psychol Women Q. (2024) 48:133–46.

[17] Endalamaw A Gilks CF Ambaw F Shiferaw WS Assefa Y. Explaining inequity in knowledge, attitude, and services related to HIV/AIDS: a systematic review. BMC Public Health. (2024) 24:1815. doi: 10.1186/S12889-024-19329-5 38978024 PMC11229290

[18] Karibayeva I Turdaliyeva B Ramazanova M. HIV prevention and awareness among people with substance use disorders in Kazakhstan: a systematic review. Journal of Clinical Medicine of Kazakhstan. (2025) 22:16–28. doi: 10.23950/JCMK/16329

[19] Hansson M Stockfelt L Urazalin M Ahlm C Andersson R. HIV/AIDS awareness and risk behavior among students in Semey, Kazakhstan: a cross-sectional survey. BMC Int Health Hum Rights. (2008) 8:1–10. doi: 10.1186/1472-698X-8-14/FIGURES/4 19087297 PMC2630293

[20] Dessie ZG Zewotir T. HIV-related stigma and associated factors: a systematic review and meta-analysis. Front Public Health. (2024) 12:1356430. doi: 10.3389/FPUBH.2024.1356430/BIBTEX 39109161 PMC11300231

[21] Endalamaw A Gilks CF Ambaw F Shiferaw WS Assefa Y. Explaining inequity in knowledge, attitude, and services related to HIV/AIDS: a systematic review. BMC Public Health. (2024) 24:1–16. doi: 10.1186/S12889-024-19329-5/FIGURES/3 38978024 PMC11229290

[22] Nakato A Kalyango JN Makoha C Orishaba P Ayebale A Kitaka SB. Knowledge on HIV and its association with sexual behavior among adolescents in Kampala, Uganda. Afr Health Sci. (2025) 25:227. doi: 10.4314/AHS.V25I2.28 PMC12361937 40837638

[23] Zhang Y Jin J Cheng F Zhang X Xu J. Impact of knowledge access on risky sexual Behaviors among Chinese youth: a pathway to improved HIV prevention (preprint). JMIR Public Health Surveill. (2024) 11:e68339. doi: 10.2196/68339 PMC12396772 40882188

[24] Glanz K Bishop DB. The role of behavioral science theory in development and implementation of public health interventions. Annu Rev Public Health. (2010) 31:399–418. doi: 10.1146/annurev.publhealth.012809.103604 20070207

[25] Rosenstock IM. The health belief model and preventive health behavior. Health Educ Behav. (1977) 2:354–86. doi: 10.1177/109019817400200405

[26] Marmot M Friel S Bell R Houweling TA Taylor S. Closing the gap in a generation: health equity through action on the social determinants of health. Lancet. (2008) 372:1661–9. doi: 10.1016/S0140-6736(08)61690-6 18994664

[27] Shaboltas AV. Psychological foundations of HIV prevention. Appendix 5 Questionnaire for Assessing Behavioral Health Risks among People Who Inject Drugs and Members of Their Social Networks. (2022):1–27. doi: 10.21638/11701/9785288062360

[28] Minina AP Turdaliyeva BS Ramazanova MA Baisugurova VY Aimbetova GY Yusupova NS. Questionnaire for studying the risks of HIV transmission among users of novel psychoactive substances (NPS). (2025)

[29] Posit team. RStudio: integrated development environment for R. Posit Software, PBC. (2023) http://www.posit.co/

[30] Chimukuche RS Shandu L Zulu S Khanyile P Singh N Gaffoor Z . HIV risk perception, trust and PrEP adherence among participants in an HIV prevention trial: a qualitative longitudinal study. South Africa *BMJ Open*. (2025) 15:e086742. doi: 10.1136/bmjopen-2024-086742 PMC12020760 40268488

[31] Loeb TA Willis K Roach MAE Sing’oei V Otieno J Oyugi R . HIV knowledge, information sources, and perceived risk among reproductive-aged individuals in Kisumu, Kenya: a latent profile analysis. AIDS Behav. (2026) 30:282–90. doi: 10.1007/s10461-025-04866-w 40897926 PMC12856736

[32] Yu B Wang Y Chen X. Perception of peer condom use buffers the associations between HIV knowledge, self-efficacy, and condom-use intention among adolescents: a moderated mediation model. Prev Sci. (2022) 23:879–88. doi: 10.1007/s11121-021-01324-6 34962622

[33] Seif SJ Oguma ED Joho AA. Using health belief model to assess the determinants of HIV/AIDS prevention behavior among university students in central, Tanzania: a cross-sectional study. PLOS Global Public Health. (2025) 5:e0004305. doi: 10.1371/journal.pgph.0004305 40009612 PMC11864508

[34] Parczewski M Gökengin D Klepikov Supplement Editors A Rossi A Biru L. The HIV epidemic in eastern Europe and Central Asia: challenges and opportunities. J Int AIDS Soc. (2024) 27:e26325. doi: 10.1002/jia2.26325 39021069 PMC11255029

[35] Neuenschwander P Altice FL Remien RH Mergenova G Sarsembayeva L Rozental E . A qualitative dyad analysis of barriers and facilitators of antiretroviral therapy (ART) adherence among people who inject drugs (PWID) with HIV in Kazakhstan. AIDS Care. (2025) 37:151–60. doi: 10.1080/09540121.2024.2414078 39404196 PMC11682916

[36] Kussainova A. Predictors of viral suppression among people living with HIV (PLHIV) on antiretroviral treatment in Almaty, Kazakhstan. University at Albany. (2025) https://scholarsarchive.library.albany.edu/etd/241 10.1080/09540121.2026.2684008 42289873

[37] Kazakh Scientific Center Of Dermatology And Infectious Diseases. Country report, 2024. Almaty. (2024). 1–6 p. https://kncdiz.kz/files/78787.pdf

[38] Pharr JR Lough NL Ezeanolue EE. Barriers to HIV testing among young men who have sex with men (MSM): experiences from Clark County, Nevada. Glob J Health Sci. (2015) 8:9–17. doi: 10.5539/gjhs.v8n7p9 PMC4965686 26925893

[39] Rowe C Matheson T Das M DeMicco E Herbst JH Coffin PO . Correlates of recent HIV testing among substance-using men who have sex with men. Int J STD AIDS. (2017) 28:594–601. doi: 10.1177/0956462416640964 27000299

[40] Iskakova B King EJ Yucel RM DeHovitz J Nugmanova Z. “I think they are infected because of their ignorance and lack of responsibility”: a mixed-methods study on HIV-related stigma in the healthcare system in Kazakhstan. PLoS One. (2025) 20:e0331201. doi: 10.1371/JOURNAL.PONE.0331201 40892800 PMC12404490

[41] Central Asian Association of People Living with HIV. THE PEOPLE LIVING WITH HIV STIGMA INDEX PEOPLE LIVING WITH HIV STIGMA INDEX 2.0 central Asian association of PLHIV. https://www.stigmaindex.org/wp-content/uploads/2022/04/Kazakhstan

[42] Mahajan AP Sayles JN Patel VA Remien RH Sawires SR Ortiz DJ . Stigma in the HIV/AIDS epidemic: a review of the literature and recommendations for the way forward. AIDS. (2008) 22:S67. doi: 10.1097/01.AIDS.0000327438.13291.62 PMC2835402 18641472

[43] Bayisa L Wakuma B Abera T Mulisa D Mosisa G Tolossa T . Are the things told to care providers kept confidential?: perceived breaches of confidentiality and associated factors among HIV/AIDS clients on ART at Nekemte specialized hospital, Western Ethiopia, 2021. HIV AIDS (Auckl). (2022) 14:1–12. doi: 10.2147/HIV.S350091 35068941 PMC8769208

[44] Lo Hog Tian JM Watson JR Ibáñez-Carrasco F Tran B Parsons JA Maunder RG . Impact of experienced HIV stigma on health is mediated by internalized stigma and depression: results from the people living with HIV stigma index in Ontario. BMC Public Health. (2021) 21:1–10. doi: 10.1186/S12889-021-11596-W/TABLES/3 34496825 PMC8427956

[45] Hutchinson P Dhairyawan R. Shame, stigma, HIV: philosophical reflections. Med Humanit. (2017) 43:225–30. doi: 10.1136/MEDHUM-2016-011179 28790137 PMC5739830

[46] Móró L. “Harm reduction of novel psychoactive substance use “In: Potter G Wouters M Fountain J, editors. Change and continuity - researching evolving drug landscapes in Europe: Pabst Publishers at European Society for Social Drug Research (2014) http://www.psychologie-aktuell.com/buecher/einzelansicht/article////1413204006-change-and-continuity-researching-evolving-drug-landscapes-in-europe.html?cHash=6f833fbe47

[47] Committee on Educating Health Professionals to Address the Social Determinants of Health. “Board on Global Health; Institute of Medicine; National Academies of sciences E and M. “Frameworks for addressing the social determinants of health” “In: A framework for educating health professionals to address the social determinants of health. Washington, DC: National Academies Press (US) (2016) https://www.ncbi.nlm.nih.gov/books/NBK395979/27854400

[48] Agency for Strategic planning and reforms of the Republic of Kazakhstan Bureau of National statistics. Prevalence of HIV infection among women and men (aged 15-49 years). Health Protection. (2024) https://gender.stat.gov.kz/page/frontend/detail?id=114&slug=15-49

[49] UNAIDS. HIV sustainability planning: Analytical resource, Kazakshtan. (2024). 1–2. https://sustainability.unaids.org/wp-content/uploads/2024/06/Kazakhstan-Executive-Summary

[50] The Global Fund. Allocation of HIV resources towards maximizing the impact of funding in selected eastern European and central Asian countries: Kazakhstan. (2023). 1–34 p. https://optimamodel.com/pubs/KAZ_HIV_2023_v2.pdf

