# Supplementary material for: Perceived HIV knowledge in the context of novel psychoactive substance use: evidence from a multi-city survey in Kazakhstan
Source: Front Public Health. 2026 Mar 10;14:1774432. doi: 10.3389/fpubh.2026.1774432 (PMC13008960; doi:10.3389/fpubh.2026.1774432)
Supplement: Supplementary file 1 [file Table_1.docx]

Supplementary Material

**Table S1**. Structural, sexual, and substance use characteristics of study participants by perceived HIV knowledge.

| **Characteristic** | | **Not informed, n (%)** | **Not enough, n (%)** | **Well informed, n (%)** | **χ²** | **p** |
| --- | --- | --- | --- | --- | --- | --- |
| **Structural characteristics** | | | | | | |
| Living conditions | Rent house/apartment | 23 (18) | 55 (27.6) | 116 (27.4) | 13.0 | 0.043 |
|  | Own house/apartment | 77 (60.2) | 98 (49.2) | 211 (49.9) |  |  |
|  | Cohabitate with relatives | 24 (18.8) | 30 (15.1) | 56 (13.2) |  |  |
|  | Cohabitate with not relatives, homeless or other | 4 (3.1) | 16 (8) | 40 (9.5) |  |  |
| Income source | Wages | 75 (58.6) | 72 (36.2) | 189 (44.7) | 20.4 | 0.009 |
|  | Social payment or support | 8 (6.2) | 14 (7) | 30 (7.1) |  |  |
|  | Illegal income | 26 (20.3) | 62 (31.2) | 108 (25.5) |  |  |
|  | Family/friends/partner | 10 (7.8) | 13 (6.5) | 31 (7.3) |  |  |
|  | Begging or other | 9 (7) | 38 (19.1) | 65 (15.4) |  |  |
| Employment | Full time | 57 (44.5) | 54 (27.1) | 121 (28.6) | 17.5 | 0.001 |
|  | Part time | 27 (21.1) | 64 (32.2) | 153 (36.2) |  |  |
|  | Unemployed | 44 (34.4) | 81 (40.7) | 149 (35.2) |  |  |
| Income level | less than 50 000 | 16 (12.5) | 37 (18.6) | 79 (18.7) | 28.2 | <0.001 |
|  | 50 000 - 100 000 | 29 (22.7) | 69 (34.7) | 161 (38.1) |  |  |
|  | 100 000 – 200 000 | 21 (16.4) | 36 (18.1) | 79 (18.7) |  |  |
|  | 200 000 and more | 62 (48.4) | 57 (28.6) | 104 (24.6) |  |  |
| Detention history | Yes | 55 (43) | 72 (36.2) | 173 (40.9) | 1.8 | 0.402 |
|  | No | 73 (57) | 127 (63.8) | 250 (59.1) |  |  |
| Use of health services | Outpatient public | 66 (51.6) | 83 (41.7) | 182 (43.0) | 3.6 | 0.169 |
|  | Hospital | 23 (18.0) | 49 (24.6) | 110 (26.0) | 3.5 | 0.1763 |
|  | Outpatient private | 12 (9.4) | 22 (11.1) | 45 (10.6) | 0.2 | 0.8848 |
|  | HIV prevention centers | 34 (26.6) | 44 (22.1) | 119 (28.1) | 2.5 | 0.2807 |
|  | NGO | 20 (15.6) | 33 (16.6) | 45 (10.6) | 5.1 | 0.0781 |
| **Sexual behavior characteristics** | | | | | | |
| Number of sexual partners (median (min - max) | | 0 (0-30) | 2 (0-50) | 2 (0-100) | 151.6 | <0.001 |
| Paid sex (last 3 months) | For money and drugs | 2 (1.6) | 38 (19.1) | 58 (13.7) | 21.4 | <0.001 |
|  | For food and stay | 0 (0) | 8 (4) | 29 (6.9) | 10.3 | 0.005 |
| Condom use behavior for paid sex | Every time | 1 (0.8) | 60 (30.2) | 112 (26.5) | 135.4 | <0.001 |
|  | Sometimes | 24 (18.8) | 104 (52.3) | 142 (33.6) |  |  |
|  | Never | 103 (80.5) | 35 (17.6) | 169 (40) |  |  |
| Anal sex with not a regular partner (last 3 months) | Yes | 125 (97.7) | 176 (88.4) | 334 (79) | 29.4 | <0.001 |
| Sex with not no consent (last 3 months) | Yes | 3 (2.3) | 23 (11.6) | 89 (21) | 29.4 | <0.001 |
| Perceived partner HIV risk (last 3 months) | Yes | 2 (1.6) | 12 (6) | 28 (6.6) | 4.8 | 0.0886 |
| Chemsex use | Yes | 9 (7) | 18 (9) | 83 (19.6) | 19.3 | <0.001 |
| Perceived drug use-sexual risk association | Yes | 27 (21.1) | 131 (65.8) | 244 (57.7) | 69.2 | <0.001 |
| Reported barrier to consistent condom use | Loss of control | 58 (45.3) | 171 (85.9) | 326 (77.1) | 71.5 | <0.001 |
|  | No thoughts on HIV risk | 6 (4.7) | 84 (42.2) | 208 (49.2) | 81.9 | <0.001 |
|  | Impossible to buy condoms nearby | 3 (2.3) | 62 (31.2) | 97 (22.9) | 39.2 | <0.001 |
|  | Lack of money | 7 (5.5) | 14 (7) | 12 (2.8) | 6.1 | 0.047 |
|  | Unwillingness to show fear | 1 (0.8) | 8 (4) | 5 (1.2) | 6.9 | 0.031 |
|  | Partner unwillingness | 0 (0) | 12 (6) | 21 (5) | 7.5 | 0.023 |
| **Substance use behavior characteristics** | | | | | | |
| Injection drug use | Yes | 46 (35.9) | 78 (39.2) | 180 (42.6) | 2.0 | 0.371 |
| Drug use pattern | Marijuana use | 62 (48.4) | 74 (37.2) | 164 (38.8) | 4.7 | 0.094 |
|  | Heroin injection | 35 (27.3) | 53 (26.6) | 95 (22.5) | 2.0 | 0.367 |
|  | Heroin inhalation / peroral | 3 (2.3) | 4 (2) | 13 (3.1) | 0.6 | 0.722 |
|  | Metadon injection | 4 (3.1) | 20 (10.1) | 29 (6.9) | 5.7 | 0.056 |
|  | Metadon inhalation/peroral | 4 (3.1) | 4 (2) | 11 (2.6) | 0.4 | 0.815 |
|  | Psychostimulators injection | 10 (7.8) | 19 (9.5) | 35 (8.3) | 0.4 | 0.825 |
|  | Psychostimulators inhalation/peroral | 33 (25.8) | 36 (18.1) | 57 (13.5) | 11.0 | 0.004 |
|  | Polydrug injection | 0 (0) | 14 (7) | 43 (10.2) | 14.6 | <0.001 |
|  | Other | 1 (0.8) | 23 (11.6) | 17 (4) | 70.0 | <0.001 |
| Recency of shared syringe use (unsterilized) | Recently | 1 (0.8) | 13 (6.5) | 93 (22) |  |  |
|  | More than a month ago | 126 (98.4) | 163 (81.9) | 313 (74) |  |  |
|  | Never | 1 (0.8) | 16 (8) | 35 (8.3) | 9.0 | 0.010 |
| History of shared syringe use with known HIV-positive person | Yes | 46 (35.9) | 78 (39.2) | 180 (42.6) | 2.0 | 0.370 |

Abbreviations: HIV – human immunidefficiency virus; NGO – non governmental organizations; SD – standard deviation.

**Table S2.** Factors associated with perceived HIV knowledge: Univariable and multivariable ordinal regression results of structural characteristics

| **Characteristic** | | **OR (95 CI)** | **p-value** | **AOR (95 CI)** | **p-value** |
| --- | --- | --- | --- | --- | --- |
| **Living conditions (Ref. Cohabitate with not relatives, homeless or other)** | Rent house/apartment | 0.73 (0.40–1.32) | 0.293 |  |  |
|  | Own house/apartment | 0.54 (0.31–0.95) | 0.032 |  |  |
|  | Cohabitate with relatives | 0.47 (0.25–0.89) | 0.019 |  |  |
| Income source (Ref. Begging or other) | Wages | 0.75 (0.50–1.14) | 0.177 |  |  |
|  | Social payment or support | 0.88 (0.47–1.68) | 0.707 |  |  |
|  | Illegal income | 0.84 (0.54–1.32) | 0.456 |  |  |
|  | Family/friends/partner | 0.83 (0.44–1.57) | 0.572 |  |  |
| Employment (Ref. Full time) | Part time | 1.76 (1.23–2.50) | 0.002 |  |  |
|  | Unemployed | 1.24 (0.89–1.74) | 0.209 |  |  |
|  | Urban | 0.84 (0.48–1.46) | 0.542 |  |  |
| Income level (Ref. 100 000 – 200 000) | less than 50 000 | 1.12 (0.70–1.78) | 0.648 |  |  |
|  | 50 000 - 100 000 | 1.22 (0.81–1.84) | 0.334 |  |  |
|  | 200 000 and more | 0.57 (0.38–0.86) | 0.008 |  |  |
| Detention histrory (Ref. No) | Yes | 1.03 (0.78–1.37) | 0.828 |  |  |
| Use of health services (Ref. No) | Outpatient public | 0.85 (0.64–1.12) | 0.251 |  |  |
|  | Hospital | 1.30 (0.94–1.81) | 0.115 |  |  |
|  | Outpatient private | 1.06 (0.67–1.66) | 0.816 |  |  |
|  | HIV prevention centers | 1.19 (0.86–1.63) | 0.301 |  |  |
|  | NGO | 0.66 (0.44–0.98) | 0.039 |  |  |

Abbreviations: AOR – adjusted odds ratio; HIV – Human Immunodeficiency Virus; NGO – nongovernmental organizations; OR – odds ratio; Ref – reference.

.
